# Characteristics and Discrepancies in Acute-on-Chronic Liver Failure: Need for a Unified Definition

**DOI:** 10.1371/journal.pone.0146745

**Published:** 2016-01-20

**Authors:** Tae Yeob Kim, Do Seon Song, Hee Yeon Kim, Dong Hyun Sinn, Eileen L. Yoon, Chang Wook Kim, Young Kul Jung, Ki Tae Suk, Sang Soo Lee, Chang Hyeong Lee, Tae Hun Kim, Jeong Han Kim, Won Hyeok Choe, Hyung Joon Yim, Sung Eun Kim, Soon Koo Baik, Byung Seok Lee, Jae Young Jang, Jeong Ill Suh, Hyoung Su Kim, Seong Woo Nam, Hyeok Choon Kwon, Young Seok Kim, Sang Gyune Kim, Hee Bok Chae, Jin Mo Yang, Joo Hyun Sohn, Heon Ju Lee, Seung Ha Park, Byung Hoon Han, Eun Hee Choi, Chang H. Kim, Dong Joon Kim, Korean Acute-on-Chronic Liver Failure (KACLiF) Study Group

**Affiliations:** 1 Institute of Medical Science, Hanyang University, Seoul, Republic of Korea; 2 Department of Internal Medicine, College of Medicine, The Catholic University of Korea, Seoul, Republic of Korea; 3 Department of Internal Medicine, Samsung Medical Center, Seoul, Republic of Korea; 4 Department of Internal Medicine, Inje University Sanggye Paik Hospital, Seoul, Republic of Korea; 5 Department of Internal Medicine, Korea University Ansan Hospital, Ansan, Republic of Korea; 6 Department of Internal Medicine, Hallym University College of Medicine, Chuncheon, Republic of Korea; 7 Department of Internal Medicine, Gyeongsang National University Hospital, Jinju, Republic of Korea; 8 Department of Internal Medicine, Catholic University of Daegu School of Medicine, Daegu, Republic of Korea; 9 Department of Internal Medicine, Ewha Womans University School of Medicine, Seoul, Republic of Korea; 10 Department of Internal Medicine, Konkuk University School of Medicine, Seoul, Republic of Korea; 11 Department of Internal Medicine, Hallym University Sacred Heart Hospital, Anyang, Republic of Korea; 12 Department of Internal Medicine, Yonsei University Wonju College of Medicine, Wonju, Republic of Korea; 13 Department of Internal Medicine, Chungnam National University, School of Medicine, Daejeon, Republic of Korea; 14 Department of Internal Medicine, Soonchunhyang University College of Medicine, Seoul, Republic of Korea; 15 Department of Internal Medicine, Dongguk University Gyeongju Hospital, Gyeongju, Republic of Korea; 16 Department of Internal Medicine, Hallym University Kangdong Sacred Heart Hospital, Seoul, Republic of Korea; 17 Department of Internal medicine, National Medical Center, Seoul, Republic of Korea; 18 Department of Internal Medicine, Soonchunhyang University Bucheon Hospital, Bucheon, Republic of Korea; 19 Department of Internal medicine, College of Medicine and Medical Research Institute, Chungbuk National University, Cheongju, Republic of Korea; 20 Department of Internal Medicine, Hanyang University Guri Hospital, Guri, Republic of Korea; 21 Department of Internal Medicine, Yeungnam University College of Medicine, Daegu, Republic of Korea; 22 Department of Internal Medicine, Inje University Haeundae Paik-Hospital, Inje University College of Medicine, Busan, Republic of Korea; 23 Department of Internal Medicine, Kosin University College of Medicine, Busan, Republic of Korea; 24 Institute of Lifestyle Medicine, Yonsei University Wonju College of Medicine, Wonju, Republic of Korea; 25 Department of Internal Medicine, University Hospitals Case Medical Center, Cleveland, Ohio, United States of America; Chiba University, Graduate School of Medicine, JAPAN

## Abstract

**Background & Aim:**

To investigate the prevalence, mortalities, and patient characteristics of Acute-on-chronic liver failure (ACLF) according to the AARC (Asian Pacific Association for the Study of the Liver ACLF Research Consortium) and European Association for the Study of the Liver CLIF-C (Chronic Liver Failure Consortium) definitions.

**Methods:**

We collected retrospective data for 1470 hospitalized patients with chronic liver disease (CLD) and acute deterioration between January 2013 and December 2013 from 21 university hospitals in Korea.

**Results:**

Of the patients assessed, the prevalence of ACLF based on the AARC and CLIF-C definitions was 9.5% and 18.6%, respectively. The 28-day and 90-day mortality rates were higher in patients with ACLF than in those without ACLF. Patients who only met the CLIF-C definition had significantly lower 28-day and 90-day survival rates than those who only met the AARC definition (68.0% vs. 93.9%, *P*<0.001; 55.1% vs. 92.4%, *P*<0.001). Among the patients who had non-cirrhotic CLD, the 90-day mortality of the patients with ACLF was higher than of those without ACLF, although not significant (33.3% vs. 6.0%, *P* = 0.192). Patients with previous acute decompensation (AD) within 1- year had a lower 90-day survival rate than those with AD more than 1 year prior or without previous AD (81.0% vs. 91.9% or 89.4%, respectively, all *P*<0.001). Patients who had extra-hepatic organ failure without liver failure had a similar 90-day survival rate to those who had liver failure as a prerequisite (57.0% vs. 60.6%, *P* = 0.391).

**Conclusions:**

The two ACLF definitions result in differences in mortality and patient characteristics among ACLF patients. We suggest that non-cirrhotic CLD, previous AD within 1 year, and extra-hepatic organ failure should be included in the ACLF diagnostic criteria. In addition, further studies are necessary to develop a universal definition of ACLF.

## Introduction

Cirrhosis is often clinically silent until decompensation occurs. Once a patient progresses to the decompensated phase, complications tend to accumulate and survival is markedly reduced. Episodes of acute deterioration due to acute insults are common causes of hospitalization among patients with chronic liver disease (CLD). However, CLD is a heterogeneous entity with different clinical presentations and variable prognosis. Recently, the concept of acute-on-chronic liver failure (ACLF) has emerged to identify those patients with CLD or cirrhosis who exhibit acute deterioration of liver function[1]. These patients are characterized by a short-term mortality rate higher than that expected for decompensated cirrhosis, with rapid progression to other end organ failure[2]. Even so, ACLF is thought to have a reversible component, with potential for full recovery[2].

Until now, ACLF has been defined variously in each study[3]. Moreover, current definitions of ACLF differ between Eastern (Asian Pacific Association for the Study of the Liver [APASL] ACLF Research Consortium, AARC) and Western countries (European Association for the Study of the Liver [EASL]-Chronic Liver Failure Consortium, CLIF-C)[4–6]. Although there are no universally accepted diagnostic criteria for ACLF, two representative definitions are commonly used. The first was proposed in 2009 by the APASL[4] and recently revised in 2014 by the AARC[5]. Later, the CLIF-C performed the EASL-CLIF acute-on-chronic liver failure in cirrhosis (CANONIC) study, which was designed to develop a definition of ACLF that is able to identify cirrhotic patients with a high risk of short-term mortality[6]. The CLIF-C proposed diagnostic criteria of ACLF are based on CLIF-sequential organ failure assessment (CLIF-SOFA) score[6]. In addition, CLIF-C developed two scoring systems, CLIF-C ACLFs (CLIF-C score for ACLF patients) and CLIF-C ADs (CLIF-C score for AD patients), to accurately predict mortality in patients with ACLF and without ACLF, respectively[7, 8].

The definitions of ACLF differ between Eastern (AARC) and Western countries (CLIF-C) in terms of CLD (confinement to liver cirrhosis only vs. encompassing liver cirrhosis and other CLD), prior AD (confinement to first AD vs. encompassing previous AD), and organ failure (liver failure as a prerequisite vs. encompassing extrahepatic organ failures)[2, 9, 10]. However, few studies have focused on the differences between the two definitions of ACLF and the resulting discrepancies in prevalence, mortality, and patient characteristics. The Korean Acute-on-Chronic Liver Failure (KACLiF) study was conducted to investigate the differences in prevalence, short-term mortality, and characteristics of ACLF patients according to the AARC and CLIF-C definitions. In addition, we investigated the impact of each definition component on short-term mortality.

## Patients and Methods

### Patients

**Fig 1:**
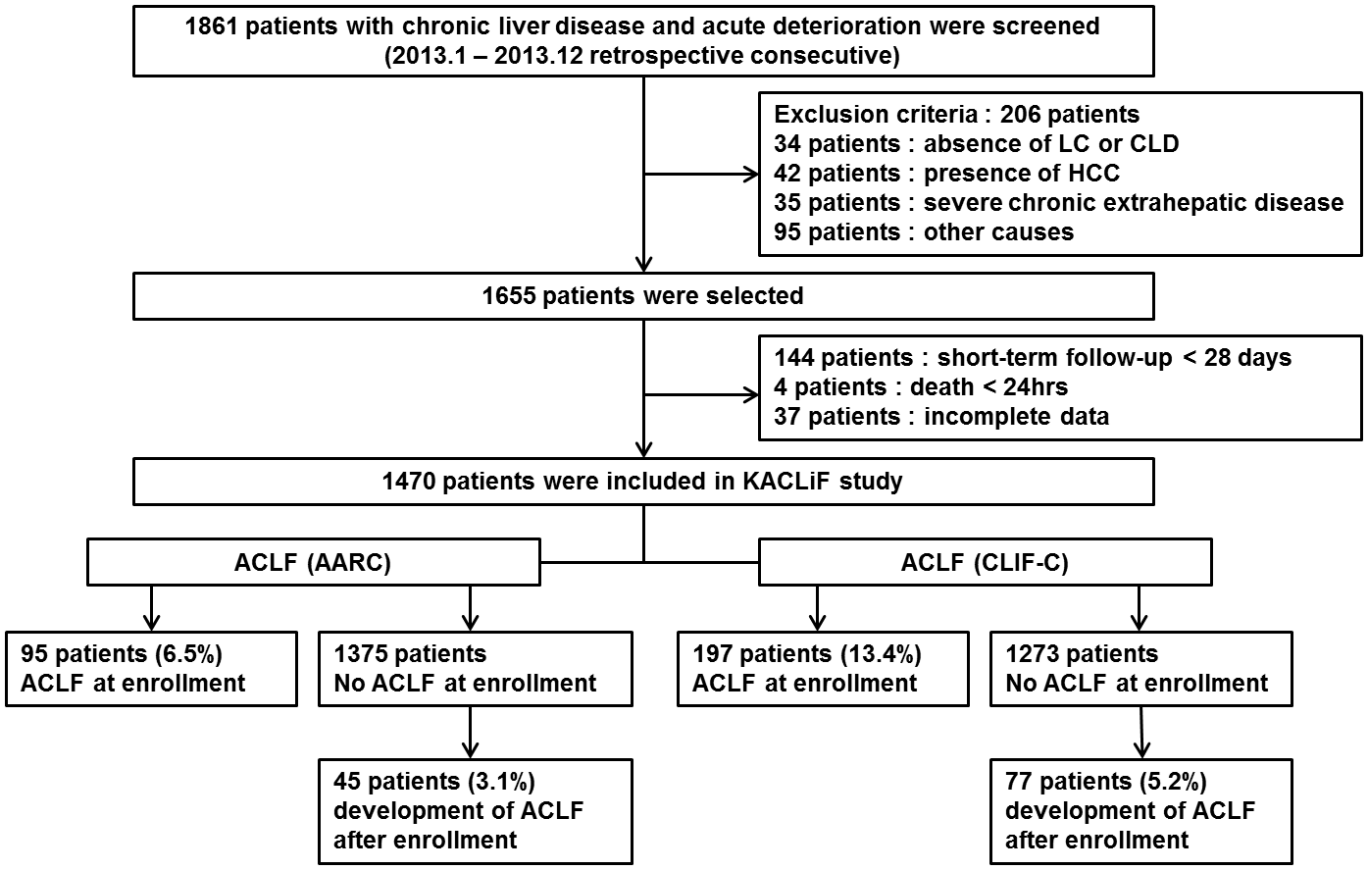
Flow chart of the KACLiF study. Abbreviations: HCC, hepatocellular carcinoma; KACLiF, Korean acute-on-chronic liver failure.

A total of 1861 patients with CLD and acute deterioration who were admitted to 21 academic hospitals were consecutively screened between January 2013 and December 2013. In this study, acute deterioration was defined as: acute development of overt ascites, hepatic encephalopathy (HE), gastrointestinal (GI) bleeding, infection, or liver dysfunction. These definitions of acute deterioration except for liver dysfunction were adopted from the CANONIC study[6]. We defined liver dysfunction as an acute increase in bilirubin level (≥3mg/dL)[11] to screen for ACLF in a larger number of admitted patients. Cirrhosis was diagnosed based on prior histological confirmation or clinical, imaging, and biochemical parameters[12]. Exclusion criteria were as follows: (1) age < 18 years, (2) absence of any CLD, (3) presence of hepatocellular carcinoma, (4) presence of severe chronic extra-hepatic disease, (5) admission due to other chronic illness, (6) human immunodeficiency virus infection, (7) chronic decompensation of end-stage liver disease, (8) less than 28 days of follow-up, and (9) incomplete data. A total of 1470 patients were analyzed (Fig 1). Follow-up continued until June 30, 2014. This study was performed in accordance with the ethical guidelines of the Declaration of Helsinki. Informed consent was not obtained, because de-identified data were analyzed. This study was approved by the Institutional Review Board of each participating hospital including Hanyang University Guri Hospital, St. Vincent’s Hospital, Uijeongbu St. Mary’s Hospital, Inje University Sanggye Paik Hospital, Korea University Ansan Hospital, Hallym University Chuncheon Sacred Heart Hospital, Gyeongsang National University Hospital, Daegu Catholic University Medical Center, Ewha Womans University Mokdong Hospital, Konkuk University Medical Center, Hallym University Sacred Heart Hospital, Wonju Severance Christian Hospital, Chungnam National University Hospital, Soonchunhyang University Hospital, Dongguk University Gyeongju Hospital, National Medical Center, Soonchunhyang University Bucheon Hospital, Chungbuk National University Hospital, Yeungnam University Medical Center, Inje University Haeundae Paik-Hospital, and Gosin University Gospel Hospital.

### Data collection and definition of clinical parameters

Data were collected on patient demographics, etiology of liver disease, clinical and laboratory variables, types of acute deterioration events, presence of organ failure, and development of ACLF. Laboratory data within 24 hours of admission and at the time of ACLF were reviewed.

AD events were classified as acute development of overt ascites, hepatic encephalopathy, GI bleeding, or infection, based on the CANONIC study[6]. Prior decompensation was defined based on the AARC definition: known previous jaundice, HE, or ascites[5]. Potential precipitating events included bacterial infection, gastrointestinal hemorrhage, active alcoholism, reactivation of underlying viral hepatitis, toxic liver injury, and others. Active alcoholism was defined as more than 21 drinks per week in men and more than 14 drinks per week in women within 3 months prior to admission[13]. If a patient was admitted with acute deterioration more than once during the observation period, data from the first admission were used in this study. Organ failure was defined according to the CLIF-SOFA score[6].

Systemic inflammatory response syndrome (SIRS) was evaluated according to the criteria of the American College of Chest Physicians/Society of Critical Care Medicine[14]. The Child-Turcotte-Pugh (CTP) score, Model for End-Stage Liver Disease (MELD) score, serum sodium (Na) to MELD score (MELD-Na)[15], and CLIF-SOFA score[6] were calculated based on the clinical variables within 24 hours of admission.

The AARC definition of ACLF was acute hepatic insult manifesting as jaundice (serum bilirubin ≥ 5mg/dL) and coagulopathy (international normalized ratio ≥ 1.5 or prothrombin time≤40%) complicated within 4 weeks by ascites and/or encephalopathy in a patient with evidence of CLD and no prior decompensation[4, 5]. The CLIF-C diagnostic criteria of ACLF were from the CANONIC study[6]. The patients with the occurrence of AD and organ failure as defined by the CLIF-SOFA score were classified as ACLF according to CLIF-C definition. ACLF development was defined as the occurrence of ACLF at or within 28 days of admission.

The primary endpoint of this study was to detect any differences in 28- and 90-day mortality according to the AARC and CLIF-C definitions. The secondary endpoints were to detect differences in mortality based on the discrepancies in the two definitions: confinement to liver cirrhosis only vs. encompassing non-cirrhotic CLD, confinement to first AD vs. encompassing previous AD, and liver failure as a prerequisite vs. encompassing extra-hepatic organ failures.

### Statistical analysis

Descriptive statistics were calculated for demographic, clinical, and laboratory characteristics. Quantitative and qualitative variables were expressed as mean±SD and number (%), respectively. Categorical variables were compared using the Chi-square test or Fisher’s exact test, and continuous variables were compared using Student’s *t*-test. The Kaplan-Meier method with log-rank test was used to calculate survival. The characteristics of discordance between the AARC and CLIF-C definitions were compared using the Chi-square test or a one-way ANOVA, and Scheffe’s post-hoc test, when appropriate. *P* value less than 0.05 was considered to be statistically significant. Statistical analysis was performed using SPSS 18.0 (SPSS, Inc. an IBM Company, Chicago, IL, USA).

## Results

### Baseline characteristics according to the definitions of AARC and CLIF-C

Baseline characteristics of the 1470 patients (1092 males, mean age 55±12 years) with acute deterioration and CLD were analyzed. The most common etiology of CLD was alcohol use (63.1%). The most common etiology of ACLF based on the definition by AARC or CLIF-C was also alcohol use (82.1% and 73.6%, respectively). Main forms of acute deterioration were gastrointestinal bleeding (GIB) (40.7%) and ascites (33.0%). Differences in baseline characteristics are summarized in Table 1.

**Table 1:** Baseline Patients Characteristics at Enrollment. ACLF was defined by the AARC or CLIF-C.

		AARC			CLIF-C		
Characteristics	All Patients (N = 1470)	No ACLF (N = 1375)	ACLF (N = 95)	*P* value	No ACLF (N = 1273)	ACLF (N = 197)	*P* value
Age (y)	55 ± 12	56 ± 12	50 ± 8	<0.001	55 ± 12	55 ± 11	0.632
Male sex	1092 (74.3)	1021 (74.3)	71 (74.7)	0.917	938 (73.7)	154 (78.2)	0.180
Presence of Cirrhosis	1352 (92.0)	1257 (91.4)	95 (100)	0.001	1155 (90.7)	197 (100.0)	<0.001
Etiology of CLD				0.009			0.068
HBV	214 (14.6)	209 (15.2)	5 (5.3)		195 (15.3)	19 (9.6)	
HCV	75 (5.1)	74 (5.4)	1 (1.1)		66 (5.2)	9 (4.6)	
HBV+HCV	2 (0.1)	2 (0.1)	0 (0.0)		2 (0.2)	0 (0.0)	
Alcohol	928 (63.1)	850 (61.8)	78 (82.1)		783 (61.5)	145 (73.6)	
HBV+alcohol	108 (7.3)	103 (7.5)	5 (5.3)		96 (7.5)	12 (6.1)	
HCV+alcohol	25 (1.7)	24 (1.7)	1 (1.1)		23 (1.8)	2 (1.5)	
Others	118 (8.0)	113 (8.2)	5 (5.3)		108 (8.5)	10 (5.1)	
Acute Decompensation^#^							
Ascites	485 (33.0)	407 (29.6)	78 (82.1)	<0.001	421 (33.1)	64 (32.5)	0.871
Hepatic encephalopathy	244 (16.6)	215 (15.6)	29 (30.5)	<0.001	169 (13.3)	75 (38.1)	<0.001
GI Bleeding	599 (40.7)	591 (43.0)	8 (8.4)	<0.001	527 (41.4)	72 (36.5)	0.197
Infection	154 (10.5)	142 (10.3)	12 (12.6)	0.478	118 (9.3)	36 (18.3)	<0.001
More than one event	150 (10.2)	127 (9.2)	23 (24.2)	<0.001	107 (8.4)	43 (21.8)	<0.001
Precipitating events	1169 (79.5)	1089 (79.2)	80 (84.2)	0.242	998 (78.4)	171 (86.8)	0.007
Bacterial infection	133 (9.0)	125 (9.1)	8 (8.4)	0.826	94 (7.4)	39 (19.8)	<0.001
GI bleeding	458 (31.2)	449 (32.7)	9 (9.5)	<0.001	398 (31.3)	60 (30.5)	0.820
Active alcoholism	595 (40.5)	531 (38.6)	64 (67.4)	<0.001	509 (40.0)	86 (43.7)	0.329
Toxic material	37 (2.5)	32 (2.3)	5 (5.3)	0.085	34 (2.7)	3 (1.5)	0.338
Reactivation of viral infection	61 (4.1)	56 (4.1)	5 (5.3)	0.590	57 (4.4)	4 (2.0)	0.109
Others	47 (3.2)	45 (3.3)	2 (2.1)	0.765	38 (3.0)	9 (4.6)	0.240
SIRS	355 (24.1)	331 (24.1)	24 (25.3)	0.793	287 (22.5)	68 (34.5)	0.001
Mean Blood Pressure (mmHg)	86 ± 17	86 ± 16	90 ± 17	0.046	88 ± 21	83 ± 21	0.003
Laboratory findings							
WBC (x10^9^/L)	8.09 ± 4.99	7.95 ± 4.91	10.08 ± 5.69	0.001	7.70 ± 4.68	10.63 ± 6.07	<0.001
ANC (x10^9^/L)	5.79 ± 4.49	5.65 ± 4.40	7.76 ±5.30	<0.001	5.39 ± 4.19	8.33 ± 5.49	<0.001
Hemoglobin (g/dL)	10.2 ± 2.8	10.2 ±2.8	10.3 ± 2.3	0.585	10.3 ± 2.8	9.2 ± 2.7	<0.001
Platelet count (x10^**9**^/L)	106 ± 63	106 ± 63	106 ± 61	0.953	108 ± 64	92 ± 53	0.001
Albumin (g/dL)	2.9 ± 0.6	2.9 ± 0.6	2.5 ± 0.5	<0.001	2.9 ± 0.6	2.5 ± 0.6	<0.001
Bilirubin (mg/dL)	5.2 ± 6.6	4.6 ± 6.1	14.2 ± 7.6	<0.001	4.5 ± 5.8	9.5 ± 9.6	<0.001
ALT (U/L)	105 ± 370	102 ± 369	144 ± 395	0.285	106 ± 382	96 ± 285	0.716
AST (U/L)	185 ± 635	164 ± 386	487 ± 2008	0.120	168 ± 393	292 ± 1418	0.226
GGT (U/L)	255 ± 368	255 ± 372	252 ± 309	0.942	267 ± 379	176 ± 274	<0.001
INR	1.53 ± 0.58	1.49 ± 0.54	2.11 ± 0.75	<0.001	1.45 ± 0.46	2.04 ± 0.93	<0.001
CRP (mg/L)	3.4 ± 9.7	3.4 ± 9.9	4.1 ± 6.7	0.465	3.0 ± 9.2	6.0 ± 12.2	0.001
Creatinine (mg/dL)	1.2 ± 1.3	1.1 ± 1.0	1.8 ± 2.9	0.019	0.9 ± 0.4	2.8 ± 2.7	<0.001
Sodium (mEq/L)	136 ± 6	136 ± 6	132 ± 7	<0.001	136 ± 6	133 ± 7	<0.001
Clinical scores							
CTP score	9 ± 2	9 ± 2	11 ± 1	<0.001	9± 2	11 ± 2	<0.001
MELD score	17 ± 7	16 ± 6	27 ± 7	<0.001	15 ± 5	27 ± 8	<0.001
MELD-Na score	19 ± 8	18± 7	29 ± 7	<0.001	17 ± 7	29 ± 7	<0.001
CLIF-SOFA score	5 ± 3	5 ± 3	9 ± 4	<0.001	4 ± 2	10 ± 4	<0.001
Organ failure by CLIF-SOFA score							
Liver	176 (12.0)	131 (9.5)	45 (47.4)	<0.001	108 (8.5)	68 (34.5)	<0.001
Kidney	137 (9.3)	114 (8.3)	23 (24.2)	<0.001	14 (1.1)	123 (62.4)	<0.001
Cerebral	104 (7.1)	87 (6.3)	17 (17.9)	<0.001	55 (4.3)	49 (24.9)	<0.001
Coagulation	78 (5.3)	58 (4.2)	20 (21.1)	<0.001	22 (1.7)	56 (28.4)	<0.001
Circulation	55 (3.7)	50 (3.6)	5 (5.3)	0.419	14 (1.1)	41 (20.8)	<0.001
Lungs	35 (2.4)	30 (2.2)	5 (5.3)	0.070	5 (0.4)	30 (15.2)	<0.001

^#^ Decompensation by the CLIF-C definition

ACLF, Acute-on-chronic liver failure; AARC, Asian Pacific Association for the Study of the Liver ACLF Research Consortium; CLIF-C, Chronic liver failure consortium; CLD, chronic liver disease; HBV, hepatitis B virus; HCV, hepatitis C virus; GI, gastrointestinal; SIRS, systemic inflammatory response syndrome; WBC, white blood cell count; ANC, absolute neutrophil count; AST, aspartate transaminase; ALT, alanine transaminase; GGT, gamma-glutamyl-transferase; INR, international normalization ratio; CRP, C-reactive protein; CTP, Child-Turcotte-Pugh; MELD, Model for End-Stage Liver Disease; CLIF-SOFA, Chronic Liver Failure—sequential organ failure assessment

### Prevalence of ACLF according to the AARC and CLIF-C

**Fig 2:**
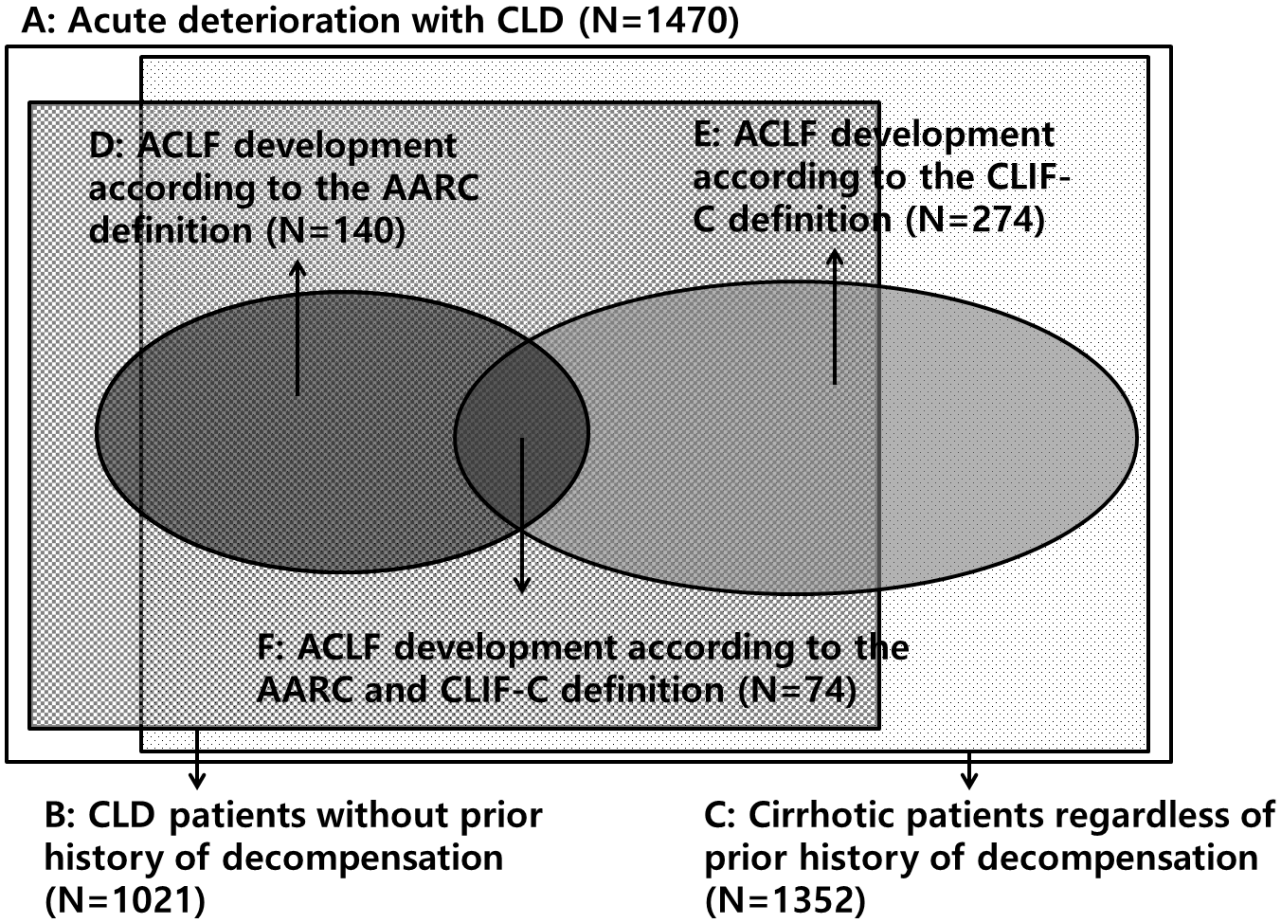
Diagram of the total enrolled patients. (A) acute deterioration with chronic liver disease (enrolled patients) (N = 1470); (B) CLD patients without prior history of decompensation (N = 1021); (C) cirrhotic patients regardless of prior history of decompensation (N = 1352); (D) ACLF development according to the AARC definition (N = 140); (E) ACLF development according to the CLIF-C definition (N = 274); (F) ACLF development according to the AARC and CLIF-C definitions (N = 74). Abbreviations: CLD, chronic liver disease; ACLF, acute-on-chronic liver failure; AARC, Asian Pacific Association for the Study of the Liver ACLF Research Consortium; CLIF-C, Chronic liver failure consortium

Of the 1470 patients, 1021 patients (69.5%) had no prior decompensation and 140 patients (9.5%) developed ACLF by the AARC definition (95 patients at admission and 45 patients within 28 days of admission). In contrast, 1352 patients (92.0%) had cirrhosis and 274 patients (18.6%) developed ACLF by the CLIF-C definitions (197 patients at admission and 77 patients within 28 days of admission). Three hundred forty patients (23.1%) met the AARC and/or CLIF-C definitions (only the AARC definition: 66 patients; only the CLIF-C definition: 200 patients; both definitions: 74 patients). ACLF developed within 28 days of admission in 45 (32.1%) and in 77 (28.1%) patients according to the AARC and CLIF-C definitions, respectively (Figs 1 and 2).

### Mortality of ACLF patients according to the AARC and/or CLIF-C definition

**Fig 3:**
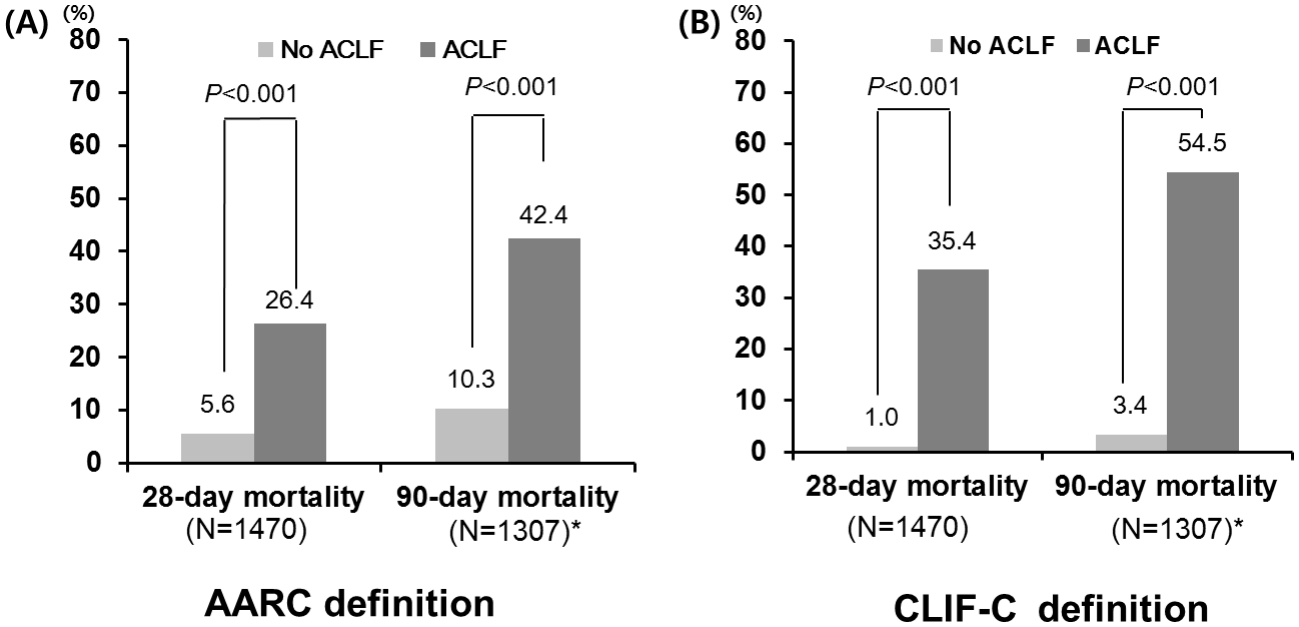
Twenty-eight- and 90-day mortality of patients with ACLF. (A) AARC definition, (B) CLIF-C definition. *One hundred sixty-three patients were lost to follow up. Abbreviations: AARC, Asian Pacific Association for the Study of the Liver ACLF Research Consortium; CLIF-C, Chronic liver failure consortium

Of the 1470 patients, 265 (18.0%) died during the follow-up period of 215±138 days. The 28-day and 90-day mortality in the study cohort were 7.6% (112/1470) and 13.2% (173/1307), respectively. The 28-day and 90-day mortality rates in patients with or without ACLF showed significant differences based on AARC and CLIF-C definition (Fig 3).

**Fig 4:**
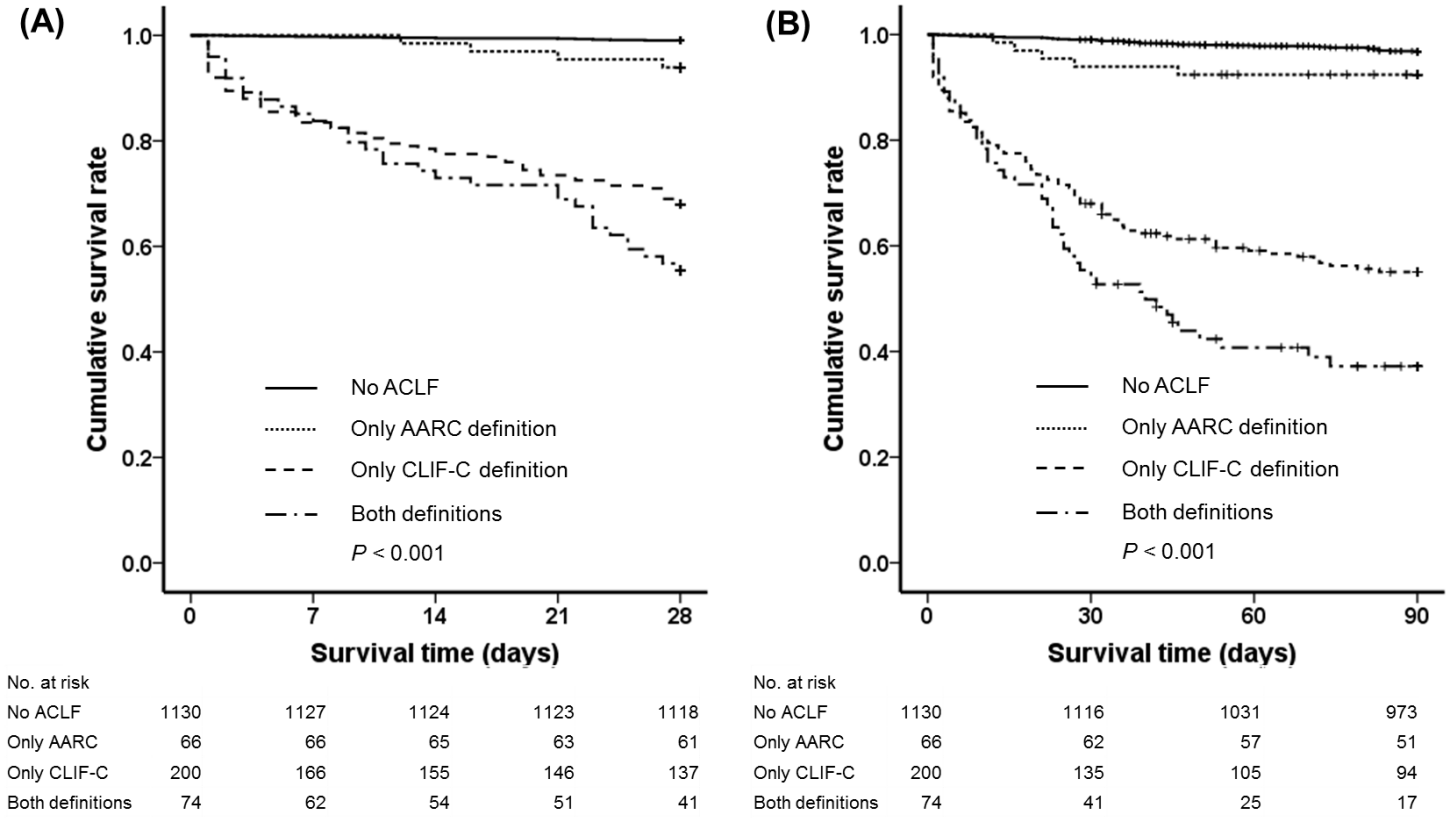
Kaplan-Meier survival curves according to the definition of ACLF. (A) 28-day survival and (B) 90-day survival. Abbreviations: ACLF, Acute-on-chronic liver failure; AARC, Asian Pacific Association for the Study of the Liver ACLF Research Consortium; CLIF-C, Chronic Liver Failure Consortium.

In patients with ACLF, the patients who satisfied both definitions showed significantly lower 28-day survival rate than those who satisfied only AARC definition (55.4% vs. 93.9%, *P* < 0.001), but not lower than those who satisfied only CLIF-C definition (55.4% vs. 68.0%, *P* = 0.081). The 90-day survival rate was significantly lower in patients who satisfied both definitions than in those who satisfied just one definition (either the AARC or the CLIF-C) (37.2% vs. 92.4% or 55.1%, *P* < 0.001) (Fig 4). Patients who only met the CLIF-C definition had significantly lower 28-day and 90-day survival rates than those who only met the AARC definition (68.0% vs. 93.9%, *P* < 0.001; 55.1% vs. 92.4%, *P* < 0.001).

**Fig 5:**
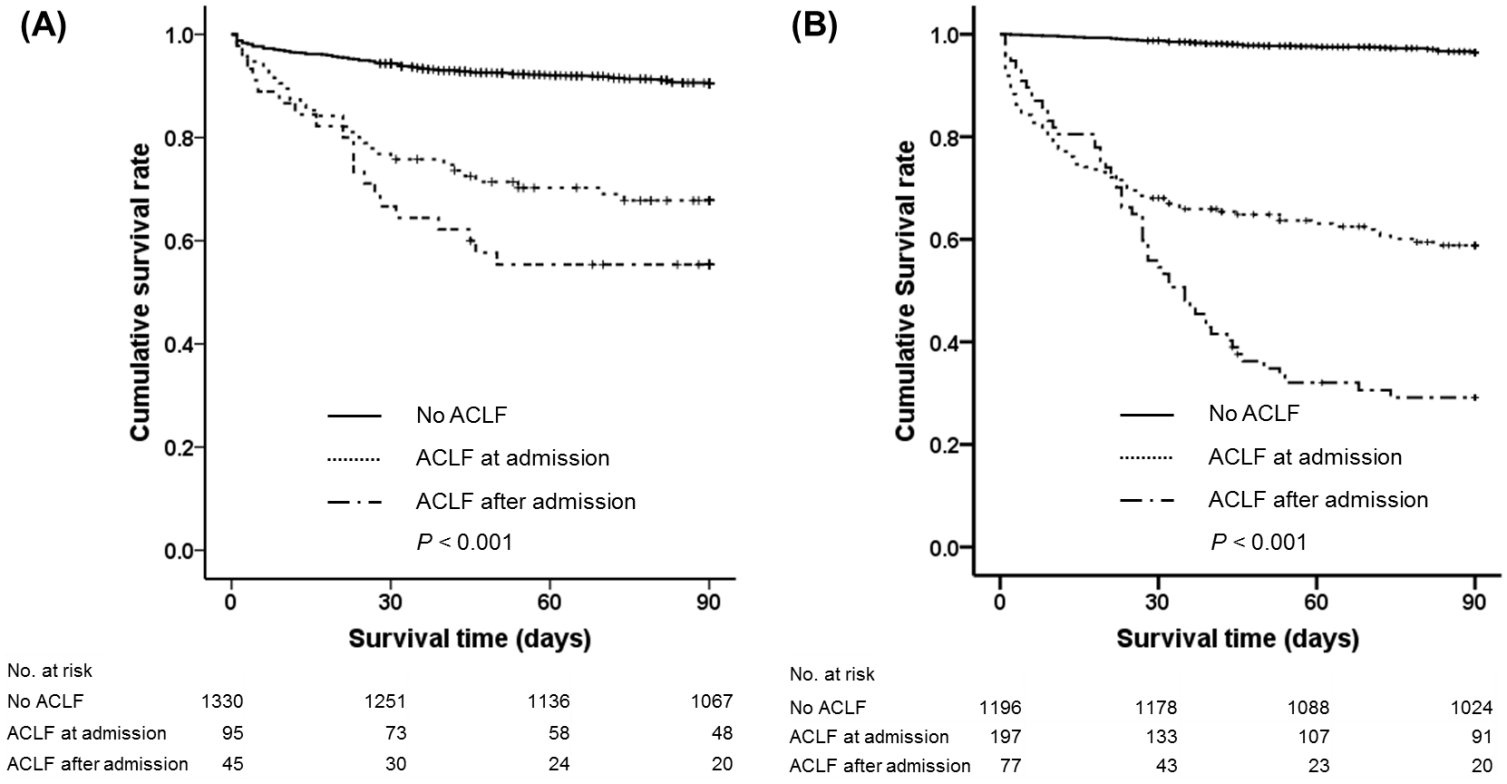
Kaplan-Meier survival curves according to the time of ACLF development. (A) AARC definition, (B) CLIF-C definition. Abbreviations: ACLF, Acute-on-chronic liver failure; AARC, Asian Pacific Association for the Study of the Liver ACLF Research Consortium; CLIF-C, Chronic liver failure consortium

Patients with ACLF at or within 28 days of admission showed a significantly lower 90-day cumulative survival rate compared to those without ACLF (according to the AARC definition: 67.8% or 55.4% vs. 90.5%, *P* < 0.001; according to the CLIF-C definition: 58.8% or 29.1% vs. 96.5%, *P* < 0.001) (Fig 5). The cumulative survival rate of those who developed ACLF after admission was significantly lower than that of those who had ACLF at admission according to the CLIF-C definition (*P* < 0.001), but not according to the AARC definition (*P* = 0.154).

### Discordant baseline characteristics between patients with ACLF defined by the AARC and CLIF-C

Baseline characteristics of patients with ACLF who met only the AARC definition, only the CLIF-C definition, or both definitions are shown in Table 2. The CLIF-C only group were older, had HE and GIB more frequently compared to the AARC only group. In contrast, ascites was more frequent as a cause of acute deterioration in the AARC only group. The CLIF-C only group had more bacterial infections and GIB, but less active alcoholism and toxic material use as the precipitating event than the AARC only group. Mean blood pressure was lower in the CLIF-C only group. In laboratory findings, the CLIF-C only group showed higher creatinine level and lower hemoglobin, and gamma-glutamyl transferase levels than the AARC only group. Patients who only met the CLIF-C definition and both definitions had more organ failure, such as kidney, cerebral, coagulation, circulation and lung failure, than patients who only met the AARC definition. In contrast, hepatic failure was more frequent in the AARC only group. In terms of clinical scoring systems, MELD, MELD-Na and CLIF-SOFA scores were higher in the CLIF-C only group than in the AARC only group.

**Table 2:** Discordant baseline characteristics between patients with ACLF defined by the AARC or CLIF-C.

Characteristics (N = 340)	AARC only (N = 66)	CLIF-C only (N = 200)	Both Definitions (N = 74)	*P* value
**Age (y)**	**50 ± 9**	56 ± 11*	**52 ± 9**^#^	**<0.001**
**Male sex**	**50 (75.8)**	**154 (77.0)**	**57 (77.0)**	**0.977**
**Cause of CLD**				**0.640**
** HBV**	**4 (6.1)**	**23 (11.5)**	**4 (5.4)**	
** HCV**	**1 (1.5)**	**12 (6.0)**	**2 (2.7)**	
** Alcohol**	**51 (77.3)**	**136 (68.0)**	**57 (77.0)**	
** HBV+alcohol**	**4 (6.1)**	**15 (7.5)**	**6 (8.1)**	
** HCV+alcohol**	**2 (3.0)**	**3 (1.5)**	**1 (1.4)**	
** Others**	**4 (6.1)**	**11 (5.5)**	**4 (5.4)**	
**Acute Decompensation**				
** Ascites**	**50 (75.8)**	58 (29.0)*	44 (59.5)*^#^	**<0.001**
** Hepatic encephalopathy**	**6 (9.1)**	63 (31.5)*	29 (39.2)*	**<0.001**
** GI Bleeding**	**8 (12.1)**	77 (38.5)*	**12 (16.2)**^#^	**<0.001**
** Infection**	**8 (12.1)**	**42 (21.0)**	**16 (21.6)**	**0.247**
** More than one event**	**8 (12.1)**	**42 (21.0)**	21 (28.4)*	**0.061**
**Precipitating events**				
** Bacterial infection**	**5 (7.6)**	37 (18.5)*	15 (20.3)*	**0.079**
** GI Bleeding**	**5 (7.6)**	62 (31.0)*	**13 (17.6)**^#^	**<0.001**
** Active alcoholism**	**43 (65.2)**	82 (41.0)*	**44 (59.5)**^#^	**0.001**
** Toxic material**	**3 (4.5)**	1 (0.5)*	**2 (2.7)**	**0.076**
** Reactivation of viral infection**	**3 (4.5)**	**5 (2.5)**	**5 (6.8)**	**0.249**
** Others**	**2 (3.0)**	**10 (5.0)**	**1 (1.4)**	**0.351**
**SIRS**	**16 (24.2)**	**65 (32.5)**	**28 (37.8)**	**0.223**
**Mean Blood Pressure (mmHg)**	**94 ± 15**	83 ± 19*	83 ± 22*	**<0.001**
**Laboratory findings**				
** WBC (x10^9^/uL)**	**9.63 ± 7.10**	**10.25 ± 6.12**	**11.88 ± 5.95**	**0.079**
** ANC (x10^9^/uL)**	**7.16 ± 6.67**	**7.99 ± 5.55**	**9.43 ± 5.31**	**0.056**
** Hemoglobin (g/dL)**	**10.8 ± 2.2**	9.3 ± 2.7*	**10.0 ± 2.6**	**<0.001**
** Platelet count (x10^9^/L)**	**110 ± 60**	**91 ± 54**	**105 ± 68**	**0.034**
** Albumin (g/dL)**	**2.6 ± 0.4**	**2.6 ± 0.6**	**2.5 ± 0.6**	**0.618**
** Bilirubin (mg/dL)**	**10.5 ± 6.8**	**8.1 ± 9.1**	15.0 ± 8.7*	**<0.001**
** ALT (U/L)**	**83 ± 144**	**65 ± 121**	**167 ± 447**^#^	**0.007**
** AST (U/L)**	**191 ± 215**	**164 ± 373**	**578 ± 2270**^#^	**0.019**
** GGT (U/L)**	**294 ± 305**	165 ± 237*	**293 ± 365**^#^	**<0.001**
** INR**	**1.71 ± 0.29**	**1.91 ± 1.08**	2.23 ± 0.97*	**0.005**
** CRP (mg/L)**	**3.76 ± 5.95**	**5.78 ± 11.93**	**4.69 ± 6.40**	**0.330**
** Creatinine (mg/dL)**	**0.9 ± 0.4**	2.4 ± 2.1*	2.3 ± 3.3*	**<0.001**
** Sodium (mEq/L)**	**135 ± 6**	**133 ± 8**	131 ± 7*	**0.006**
**Clinical scores**				
** CTP score**	**11 ± 1**	**10 ± 2**	11 ± 2*^#^	**<0.001**
** MELD score**	**21 ± 4**	24 ± 7*	28 ± 8*^#^	**<0.001**
** MELD-Na score**	**23 ± 5**	27 ± 7*	31± 7*^#^	**<0.001**
** CLIF-SOFA score**	**6 ± 1**	9 ± 4*	10 ± 4*^#^	**<0.001**
**Organ failure by CLIF-SOFA score**				
** Liver**	**16 (24.2)**	**52 (26.0)**	40 (54.1)*^#^	**<0.001**
** Kidney**	**2 (3.0)**	98 (49.0)*	27 (36.5)*	**<0.001**
** Cerebral**	**1 (1.5)**	35 (17.5)*	17 (23.0)*	**0.001**
** Coagulation**	**1 (1.5)**	42 (21.0)*	23 (31.1)*	**<0.001**
** Circulation**	**1 (1.5)**	34 (17.0)*	9 (12.2)*	**0.005**
** Lungs**	**0 (0.0)**	23 (11.5)*	8 (10.8)*	**0.016**
**Hospital days**	**22 ± 24**	**20 ± 31**	**26 ± 34**	**0.331**
**28-day mortality**	**4 (6.1)**	64(32.0)*	33 (44.6)*	**<0.001**
**90-day mortality (N = 300)** ^§^	**5 (8.9)**	88 (48.4)*	45 (72.6)*^#^	**<0.001**

*, *P* < 0.05 vs. only the AARC definition;

^#^, *P* <0.05 vs. only the CLIF-C definition.

^§^, Forty patients were lost to follow up at 90 days

ACLF, acute-on-chronic liver failure; AARC, Asian Pacific Association for the Study of the Liver ACLF Research Consortium; CLIF-C, Chronic Liver Failure Consortium; CLD, chronic liver disease; HBV, hepatitis B virus; HCV, hepatitis C virus; GI, gastrointestinal; SIRS, systemic inflammatory response syndrome; WBC, white blood cell count; ANC, absolute neutrophil count; ALT, alanine transaminase; AST, aspartate transaminase; GGT, gamma-glutamyl-transferase; INR, international normalized ratio; CRP, C-reactive protein; CTP, Child-Turcotte-Pugh; MELD, Model for End-Stage Liver Disease; CLIF-SOFA, chronic liver failure—sequential organ failure assessment

### Mortality according to the definition of underlying CLD (confinement to liver cirrhosis only vs. encompassing liver cirrhosis and other CLD)

**Fig 6:**
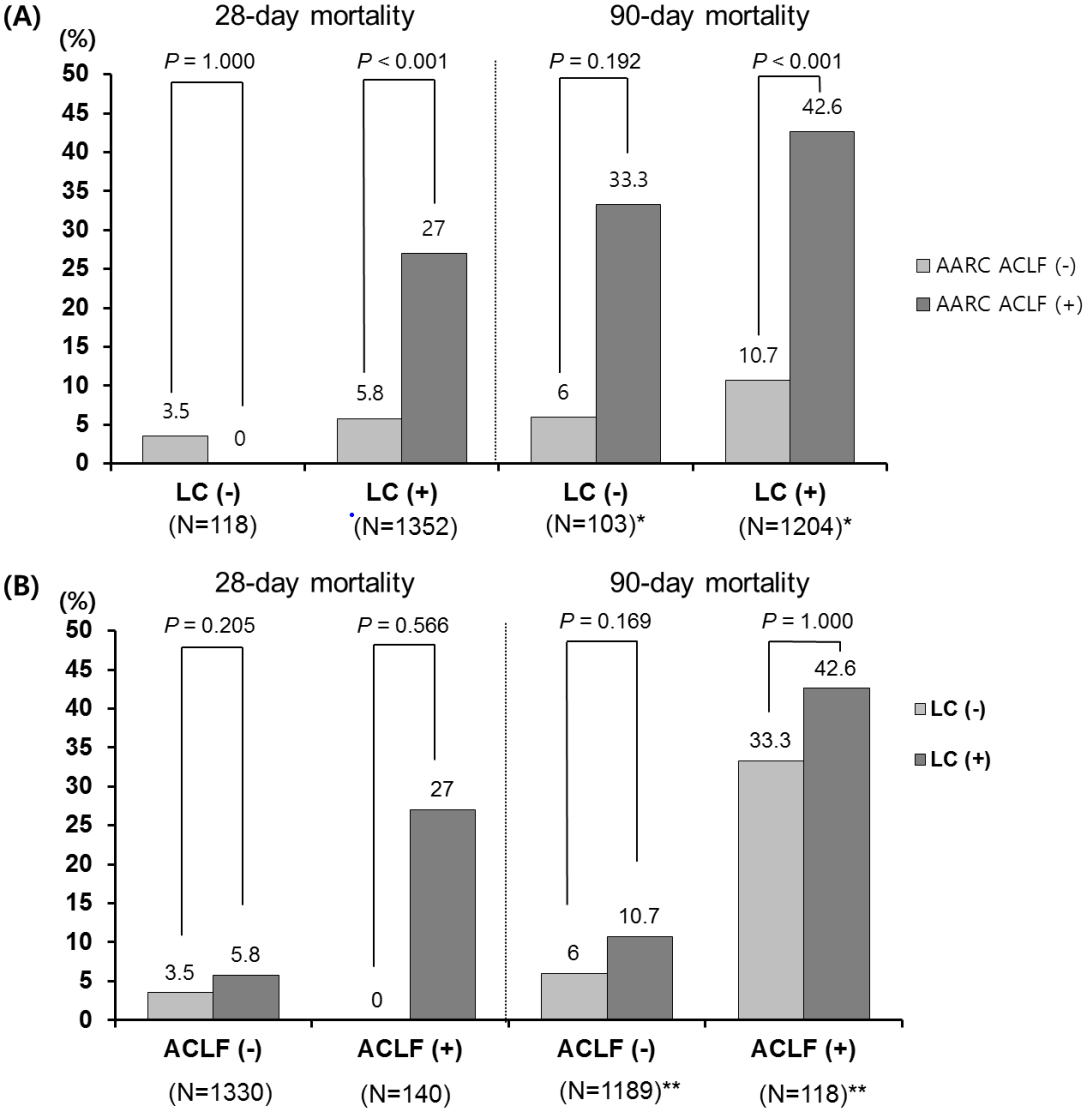
Twenty-eight- and 90-day mortality. (A) According to the presence of cirrhosis (*15 of patients without LC and 148 patients with LC were lost to follow-up) and (B) according to the presence of ACLF (**138 of patients without ACLF and 31 patients with ACLF were lost to follow-up). Abbreviations: LC, liver cirrhosis; ACLF, acute-on-chronic liver failure

We investigated whether the presence of non-cirrhotic CLD influenced mortality in total enrolled patients with acute deterioration. Because the CLIF-C defines ACLF only in those patients with liver cirrhosis, we analyzed mortality difference according to the presence of ACLF as defined by the AARC (Fig 6).

Among patients with liver cirrhosis, the 28-day and 90-day mortalities of patients with ACLF were higher than those without ACLF (28-day mortality: 27.0% vs. 5.8%, *P* < 0.001; 90-day mortality: 42.6% vs. 10.7%, *P* < 0.001). Among patients without liver cirrhosis, the 90-day mortality of patients with ACLF was, although not significant, higher than those without ACLF (33.3% vs. 6.0%, *P* = 0.192). However, there was no significant difference in the 28-day mortality (0% vs. 3.5%, *P* = 0.353) (Fig 6A).

On the other hand, there were no significant differences in the 28-day and 90-day mortality between patients without and with cirrhosis in patients without ACLF (28-day mortality: 3.5% vs. 5.8%, *P* = 0.205; 90-day mortality: 6.0% vs. 10.7%, *P* = 0.169) and with ACLF (28-day mortality: 0% vs. 27.0%, *P* = 0.566; 90-day mortality: 33.3% vs. 42.6%, *P* = 1.000) (Fig 6B).

### Mortality according to the presence of previous AD (confinement to first AD without previous AD vs. encompassing previous AD)

**Fig 7:**
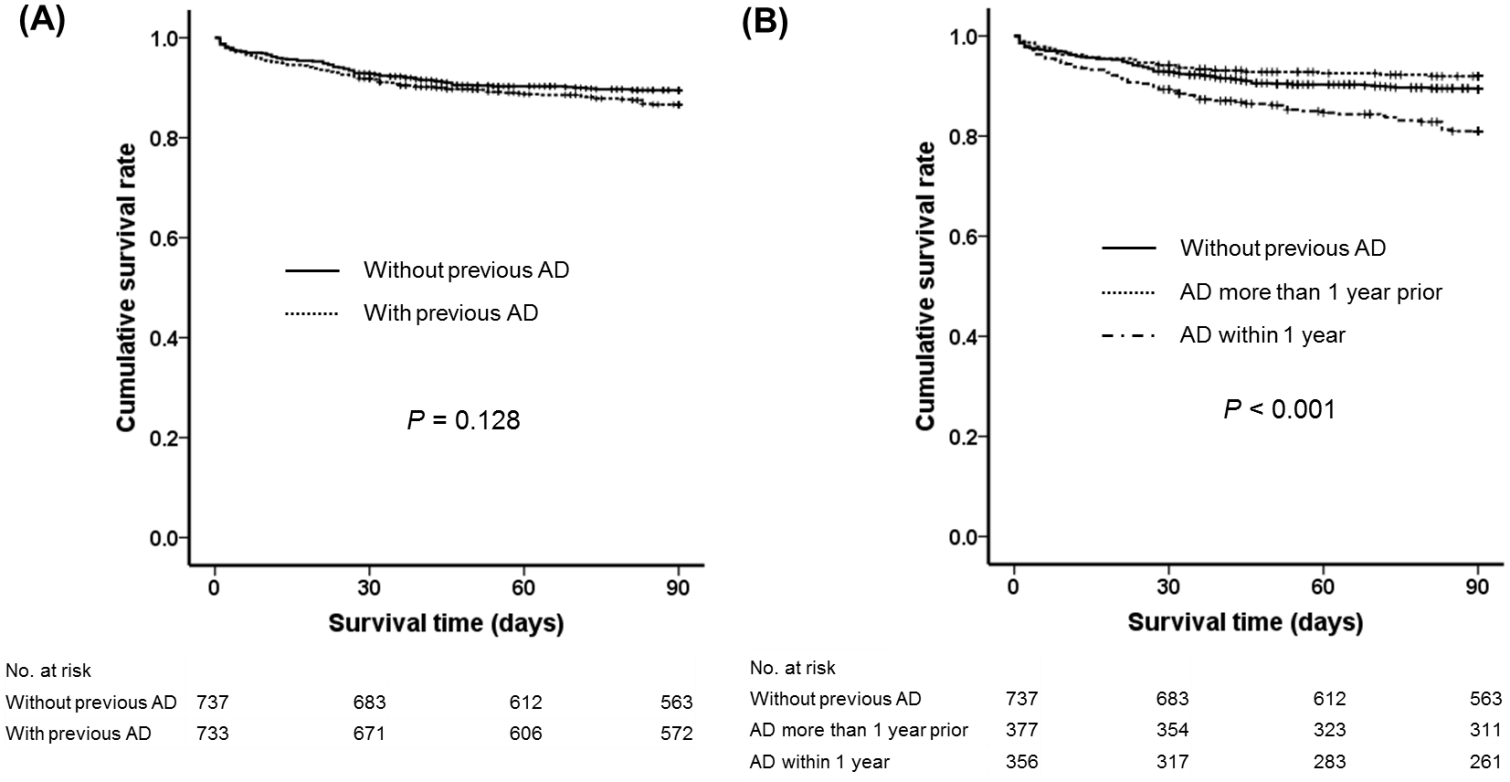
Ninety-day survival curves according to previous acute decompensation. (A) Without previous AD vs. with previous AD and (B) without previous AD vs. AD more than 1 year prior vs. AD within 1 year. Abbreviation: AD, acute decompensation

We analyzed the survival difference in patients with or without previous history of AD. Of 1470 patients with acute deterioration of CLD, 733 patients (49.9%) had been hospitalized with previous AD based on the CLIF-C definition. There was no significant difference in the cumulative survival rate between the patients with and without previous AD (86.6% vs. 89.4%, *P* = 0.128) (Fig 7A). When we divided the patients with previous AD into two groups depending on the time of previous AD (more than 1 year prior vs. within 1 year), patients with AD within 1 year showed a significantly lower survival rate than those without AD (81.0% vs. 89.4%, *P* < 0.001) and with AD more than 1 year prior (81.0% vs. 91.9%, *P* < 0.001), although no significant difference was seen between patients with AD more than 1 year prior and without AD (91.9% vs. 89.4%, *P* = 0.185) (Fig 7B).

### Mortality of ACLF patients according to the definition of organ failure (liver failure as a prerequisite vs. extra-hepatic organ failures without liver failure)

**Fig 8:**
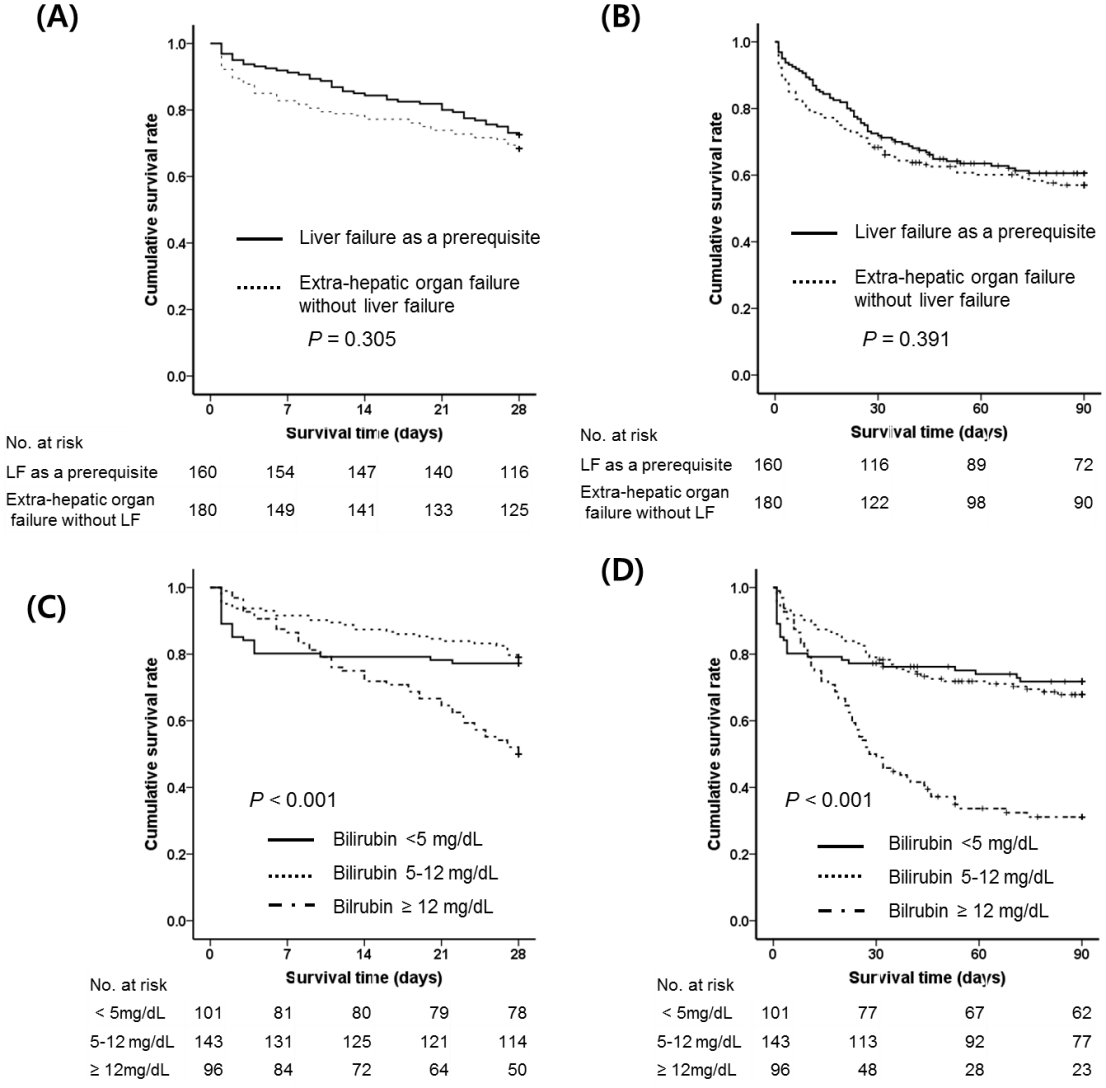
Kaplan-Meier survival curves of ACLF according to the definition of organ failure (liver failure as a prerequisite vs. extra-hepatic organ failure). (A) 28-day and (B) 90-day survival according to liver failure as defined by the AARC definition, (C) 28-day and (D) 90-day survival according to bilirubin level. Abbreviations: ACLF, acute-on-chronic liver failure; AARC, Asian Pacific Association for the Study of the Liver ACLF Research Consortium

To clarify whether liver failure is a prerequisite for defining ACLF, we analyzed the characteristics of ACLF in patients with liver failure and patients with extra-hepatic organ failures. Of the 340 patients with ACLF according to either the AARC or CLIF-C definition, we compared the 160 patients who had liver failure according to the AARC definition (bilirubin ≥ 5mg/dL and INR ≥ 1.5) to the remaining 180 patients who had extra-hepatic organ failure but without liver failure (Fig 8A and 8B). Kaplan Meier analysis showed that the 28-day and 90-day cumulative survival rates of those who had extra-hepatic organ failure without liver failure were similar to those of patients who had liver failure as a prerequisite (28-day survival: 68.3% vs. 72.5%, *P* = 0.305; 90-day survival: 57.0% vs. 60.6%, *P* = 0.391). Because the CLIF-C criterion for liver failure is bilirubin ≥ 12 mg/dL, we performed survival analysis of 3 groups divided by serum bilirubin level (group 1: < 5 mg/dL, group 2: 5–12 mg/dL, and group 3: ≥ 12 mg/dL). The 28-day and 90-day survival rates of group 3 were significantly lower than those of group 1 (50.0% vs. 77.2%, *P* = 0.001 and 31.1% vs. 71.8%, *P* < 0.001) and group 2 (50.0% vs. 79.0%, *P* < 0.001 and 31.1% vs. 67.8%, *P* < 0.001), whereas there was no significant difference between the rates of groups 1 and 2 (*P* = 0.599 and *P =* 0.726) (Fig 8C and 8D).

## Discussion

ACLF, which results in rapidly deteriorating liver function in patients with underlying CLD, is associated with poor prognosis. Eastern (AARC) and Western (CLIF-C) countries have proposed definitions of ACLF to identify these patients at a high risk of short-term mortality[5, 6]. However, the two definitions of ACLF differ from each other in many ways. This study demonstrated resultant differences in prevalence and mortality of ACLF patients according to the two definitions. In addition, we compared short-term mortality rates according to different criteria among the two definitions: predisposition (CLD vs. cirrhosis only, and first AD only vs. any previous AD) and organ dysfunction (liver failure as a prerequisite vs. extra-hepatic organ failure).

In this study, among 1470 acutely deteriorated CLD patients, the prevalence of ACLF was 9.5% vs. 18.6%, according to the AARC and CLIF-C definitions, respectively. Prevalence based on the CLIF-C definition is somewhat lower than that seen in the CANONIC study (22.6%)[6] and the single center validation study by Silva et al. (24%)[16]. This might be because of the criterion of acute deterioration. This study included jaundice (bilirubin ≥ 3 mg/dL) as acute deterioration criterion, which might have enrolled more acutely deteriorated patients without ACLF. If we included only those patients who fulfilled the AD criteria of the CANONIC study (excluding patients with only jaundice [bilirubin ≥ 3 mg/dL]), the prevalence of ACLF was 20.1%, which is similar to that of the CANONIC study.

Patients with ACLF based on both definitions showed significantly higher short-term mortality than those without ACLF (Fig 3). These findings suggest that both ACLF definitions were able to independently identify the patients with a high risk of short-term mortality. However, there was a significant difference in short-term mortality between patients with ACLF according to the CLIF-C and AARC definitions (Fig 4). The CLIF-C predefined a 28-day mortality rate greater than 15% as a threshold, whereas the AARC has taken estimated 33% mortality at 28 days into account. In this study, the 28-day and 90-day mortality rates (35.4% and 54.5%, respectively) of ACLF patients based on the CLIF-C definition satisfied the predefined mortality rate threshold and were similar to the results of the CANONIC study[6]. However, the 28-day mortality rate of ACLF patients based on the AARC definition (26.4%) did not satisfy the predefined mortality threshold, and the 28-day and 90-day mortality rates were lower than those in the AARC study[17]. In addition, even if the previous decompensation within 1 year and extrahepatic organ failure were included, the 28-day mortality rates were also lower than the predefined mortality threshold (previous decompensation within 1 year: 24.3%, extrahepatic organ failure: 26.7%) (data not shown). The low mortality rates seen in this study likely resulted from the differences in patients characteristics compared to the AARC study.

The CANONIC study showed that the mortality of patients with ACLF at admission (33.9%) was similar to that of patients who developed ACLF after admission (29.7%)[6]. However, this study showed that patients who developed ACLF after admission had a worse 90-day survival compared to those with ACLF at admission. ACLF development after admission may result from a natural disease course, but some could result from new acute insults, such as nosocomial infection, GI bleeding, or hepatotoxic medication. Therefore, although some patients with acute deterioration may not have ACLF at admission, clinicians should make an effort to prevent patient exposure to new insults, and to detect the development of ACLF early.

Bacterial infection and GIB were more frequent in ACLF patients according to the CLIF-C definition, while active alcoholism and use of toxic material were more frequent in ACLF patients according to the AARC definition in this study. These findings may result from how an acute insult is defined. Active alcohol abuse and toxic material use are typical hepatic insults, and bacterial infections and GIB are typically non-hepatic insults. While CLIF-C definition include non-hepatic insults, whether variceal hemorrhage and sepsis is included is not clear in AARC definition[5, 6]. Duseja et al. reported that non-hepatic insults are common, accounting for 60% of ACLF according to the AARC definition except precipitating events[18]. Likewise, non-hepatic insults were common in this study, accounting for 43.9% of ACLF. A previous study had reported that patients with hepatic vs. non-hepatic insults had distinct clinical features, and the non-hepatic insult group had a higher 90-day mortality[19]. In addition, infection, typically a non-hepatic insult, is known to be an independent prognostic factor[20, 21]. Therefore, considering the large proportion and high mortality rate, non-hepatic insults should be considered as important precipitating events in ACLF.

The two ACLF definitions define underlying CLD differently. This difference might be due to differences in underlying CLDs and acute insults. More patients had viral infections as underlying CLD and viral superinfections or reactivation of HBV as acute insults in the East than the West[6, 17]. Cirrhosis is not necessary for the development of liver failure by reactivation of HBV or acute viral superinfection. Even without cirrhosis, acute viral superinfections in patients with CLD presented with a more severe course and higher mortality than those without CLD[22, 23]. In this study, non-cirrhotic CLD patients with ACLF according to the AARC definition showed a higher 90-day mortality, although not statistically significant (Fig 6). In addition, the short-term mortality rates (28-day and 90-day) did not differ between two groups, regardless of the presence of ACLF. This suggests that the presence of cirrhosis per se is not associated with increased mortality in ACLF patients. Although this study included small number of non-cirrhotic patients (118 patients), because of the high 90-day mortality of the non-cirrhotic ACLF patients, it would be better to consider non-cirrhotic CLD as an underlying CLD of ACLF.

The interesting finding is that the etiologies of ACLF was changed. In the 2000’s, the main cause of underlying disease in ACLF was alcohol use in Europe[24], whereas in the Asia-Pacific region, it was hepatitis B virus[25, 26]. However, according to recent studies of Asia-Pacific region, alcohol use was the most common etiology of underlying CLD[17, 27]. Similarly, our multicenter study in Korea also found that the main cause of underlying liver disease in CLD with acute deterioration was alcohol use. These results may have come from the introduction of universal HBV vaccination program as well as the widespread application of oral antiviral therapy for HBV infection in Korea[28].

Another difference in underlying CLD between the two definitions is whether patients with previous decompensation are included or not. Patients with previous decompensation with jaundice, HE, and ascites are excluded in the AARC definition[5]. On the contrary, the CANONIC study included these patients, if it was a new AD episode[6]. In this study, there was no difference between patients with and without previous AD according to the CLIF-C definition(*P* = 0.128). However, patients who had AD within 1 year showed a significantly lower survival rate than those with AD more than 1 year prior and those without previous AD. Therefore, considering the high mortality rate, it would be better to include the patients who developed AD within 1 year in the definition of ACLF. Interestingly, these results contradict the result of the CANONIC study, which reported that the patients without previous AD had higher mortality rate than those without previous AD owing to a lack of tolerance[6]. High mortality of patients with previous AD in this study could be explained by reduced hepatic functional reserve. Patients with previous AD, especially within 1 year, are likely to have reduced hepatic functional reserve because of insufficient time for recovery. Additional acute insult may then lead to more rapid deterioration and higher mortality.

The CLIF-C places more emphasis on extrahepatic organ failure, especially kidney failure[6]. However, in the AARC definition, liver failure is mandatory regardless of extrahepatic organ failure[5]. When liver failure was defined by the AARC, there was no difference in short-term survival rate between patients who developed extrahepatic organ failure without liver failure and those who had liver failure as a prerequisite, regardless of extrahepatic organ failure. This result means that extrahepatic organ failure is important prognostic factor as much as the liver failure is. However, unlike the AARC, the CLIF-C defines liver failure as bilirubin ≥ 12 mg/dL. When liver failure was defined by the CLIF-C definition, patients with liver failure showed a lower survival rate than those without liver failure. Bilirubin ≥ 12 mg/dL was an independent predictor for short-term mortality (*P* < 0.001) and was significantly associated with more frequent cerebral, coagulation, and circulation failure compared to bilirubin < 12 mg/dL (all *P* < 0.05)(data not shown). Interestingly, patients with a bilirubin 5–12 mg/dL seemed to have better short-term survival than patients with a bilirubin < 5 mg/dL, even though not statistically significant (Fig 8). This result might be associated with other organ failure. In this study, patients with a bilirubin < 5 mg/dL had significantly more frequent kidney failure than patients with a bilirubin 5–12 mg/dL (*P* < 0.001). In other words, extra-hepatic organ failure may be important for short-term mortality as liver failure. Therefore, extrahepatic organ failure should be included as a diagnostic criterion for ACLF, and further studies are necessary to identify the optimal bilirubin cut-off level for diagnosing ACLF.

This study has several limitations. First, it was a retrospective study, which may have led to selection bias. To overcome this limitation, we consecutively enrolled subjects for the study and collected follow-up data for an average of 6 months. Second, alcohol use was the main etiology of CLD and acute insults. In addition, non-cirrhotic CLD patients accounted for only a small proportion (8.0%) of the study group. Thus, to define ACLF more accurately, prospective studies that include more diverse etiology and precipitating factors or studies individualized by etiology are necessary.

In conclusion, discrepant ACLF definitions between Eastern and Western countries resulted in differences in mortality and patient characteristics, which arise because underlying CLD, precipitating factors, and organ failures are defined differently. We suggest that non-cirrhotic CLD, previous AD within 1 year, and extrahepatic organ failure should be included in the diagnostic criteria for ACLF. Efforts are urgently needed to bridge the difference between the two definitions and to develop a universal definition of ACLF.
